# Visual Attention of Anesthesia Providers in Simulated Anesthesia Emergencies Using Conventional Number-Based and Avatar-Based Patient Monitoring: Prospective Eye-Tracking Study

**DOI:** 10.2196/35642

**Published:** 2022-03-22

**Authors:** Arsène Ljubenovic, Sadiq Said, Julia Braun, Bastian Grande, Michaela Kolbe, Donat R Spahn, Christoph B Nöthiger, David W Tscholl, Tadzio R Roche

**Affiliations:** 1 Institute of Anesthesiology University of Zurich University Hospital Zurich Zurich Switzerland; 2 Departments of Epidemiology and Biostatistics Institute of Epidemiology, Biostatistics and Prevention University of Zurich Zurich Switzerland; 3 Simulation Centre University Hospital Zurich Zurich Switzerland

**Keywords:** Anesthesia, eye-tracking technology, patient monitoring, patient simulation, situation awareness, task performance, visual attention, avatar based model, simulated anesthesia, perioperative

## Abstract

**Background:**

Inadequate situational awareness accounts for two-thirds of preventable complications in anesthesia. An essential tool for situational awareness in the perioperative setting is the patient monitor. However, the conventional monitor has several weaknesses. Avatar-based patient monitoring may address these shortcomings and promote situation awareness, a prerequisite for good decision making.

**Objective:**

The spatial distribution of visual attention is a fundamental process for achieving adequate situation awareness and thus a potential quantifiable surrogate for situation awareness. Moreover, measuring visual attention with a head-mounted eye-tracker may provide insights into usage and acceptance of the new avatar-based patient monitoring modality.

**Methods:**

This prospective eye-tracking study compared anesthesia providers' visual attention on conventional and avatar-based patient monitors during simulated critical anesthesia events. We defined visual attention, measured as fixation count and dwell time, as our primary outcome. We correlated visual attention with the potential confounders: performance in managing simulated critical anesthesia events (task performance), work experience, and profession. We used mixed linear models to analyze the results.

**Results:**

Fifty-two teams performed 156 simulations. After a manual quality check of the eye-tracking footage, we excluded 57 simulations due to technical problems and quality issues. Participants had a median of 198 (IQR 92.5-317.5) fixations on the patient monitor with a median dwell time of 30.2 (IQR 14.9-51.3) seconds. We found no significant difference in participants' visual attention when using avatar-based patient monitoring or conventional patient monitoring. However, we found that with each percentage point of better task performance, the number of fixations decreased by about 1.39 (coefficient –1.39; 95% CI –2.44 to –0.34; *P*=.02), and the dwell time diminished by 0.23 seconds (coefficient –0.23; 95% CI: –0.4 to –0.06; *P*=.01).

**Conclusions:**

Using eye tracking, we found no significant difference in visual attention when anesthesia providers used avatar-based monitoring or conventional patient monitoring in simulated critical anesthesia events. However, we identified visual attention in conjunction with task performance as a surrogate for situational awareness.

## Introduction

Continuous patient monitoring in anesthesia is well established in today’s operating theaters and described by the World Health Organization as essential to achieving safe surgical conditions [1]. However, although patient monitoring is a crucial tool for situation awareness in the perioperative setting [2-4], it has several shortcomings, mainly related to the number- and waveform-based monitoring, which may not fully exploit the capabilities of human sensory perception [5-7]. Information overload and alarm fatigue adversely affect care providers’ situation awareness [6-9], potentially leading to critical errors during anesthesia [10].

To enhance situation awareness, new approaches are explored [8,11-15], one being avatar-based patient monitoring [16]. Based on the conventional numerical monitoring data, we created the visual-patient-avatar, representing an animated virtual model of the monitored patient. The avatar abstracts the information and enables health care professionals to assess the patient's condition globally and detect subtle but consequential changes [17]. Computer-based studies found that health care professionals retrieved more vital signs with increased diagnostic confidence and reduced perceived workload when using avatar-based instead of conventional monitoring [17-19]. In addition, a high-fidelity simulation study found that the technology helped anesthesia teams to diagnose what was wrong with the patient more quickly [20]. Moreover, the technology received positive feedback from health care professionals and was rated as easy to learn and use [21,22].

Exploring how health care providers visually interact with new medical devices may provide insights into their usage and acceptance and identify areas for improvement. Furthermore, the spatial distribution of visual attention is a fundamental process for achieving adequate situation awareness [23] and thus a potential quantifiable surrogate for situation awareness [24]. A powerful tool for objectifying the visual attention between users and their environment is eye tracking [25].

Using realistically simulated critical anesthesia events, this study used eye tracking to investigate whether avatar-based patient monitoring influences anesthesia providers’ visual attention on the patient monitor. Based on the accelerated and simplified information transfer found in the previous studies [17-19,26,27], we hypothesized that the anesthesia provider's visual attention on the patient monitor would decrease when using avatar-based patient monitoring. Furthermore, we tested how several potential confounders such as work experience, profession (ie, physician or nurse anesthetist), or task performance in managing the simulated critical events influenced visual attention on the patient monitor.

## Methods

### Ethics Approval

The Cantonal Ethics Committee of Zurich, Switzerland, reviewed the study protocol and issued a declaration of no objection (Business Management System for Ethics Committees Number Req-2020-00059). We collected all data under written informed consent by all participants.

### Study Design

**Figure 1:**
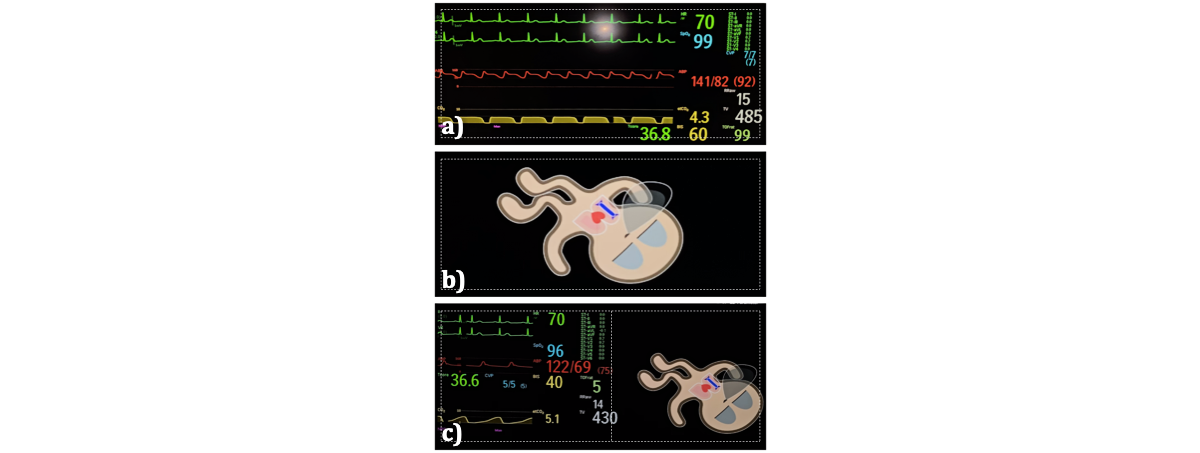
Different screen modalities used in the simulation study. a) Conventional number- and waveform-based monitoring. b) only avatar-based monitoring. c) split-screen monitoring, displaying both modalities side-by-side. White boxes indicate our area of interest on the patient monitor used for post hoc semi-automated video analysis.

This investigator-initiated, prospective, randomized, eye-tracking study investigated anesthesia providers’ visual attention on the patient monitor during simulated critical anesthesia events. We used three different patient monitor screen modalities (Figure 1). We collected all eye-tracking data during a simulation study conducted in May 2020 at the University Hospital Zurich in Switzerland [20].

### Study Procedure

We included 52 anesthesia teams consisting of a nurse anesthetist and an anesthesiologist. In randomized order, each team simulated the scenario of severe bronchospasm, myocardial infarction, and malignant hyperthermia once. Each simulated scenario lasted 10 minutes. For each scenario, the teams used a different screen modality: only conventional patient monitoring (ie, number- and waveform-based monitoring), only the avatar-based patient monitoring (visual-patient-avatar), or split-screen monitoring consisting of both modalities shown side-by-side simultaneously (Figure 1). In a randomized order, we chose one of the team members to be the team leader. During the scenarios, mentioned leader (ie, either the nurse anesthetist or the physician) wore a mobile eye-tracking device (Pupil Invisible; Pupil Labs, GmbH) while the team managed the critical incident together. We used Research Randomizer Version 4.0 [28] to randomize the order of the scenarios, respective team leaders, and screen modality.

### Simulation Environment

We conducted the study in a backup operating room with an analogous setup as the study center’s active operating rooms. To enhance the simulation fidelity, we used real medications and airway management tools in addition to a state-of-the-art, full human patient simulator (HAL S301; Gaumard Scientific Company, Inc). We used a Philips InteliVue MX500 (Koninklijke Philips NV) patient monitor to closely resemble the study center’s MX550 and MX800 monitors. In the simulation environment, we tagged the patient monitor as our area of interest using pre-generated QR codes. These QR codes enable the Pupil Player software to automatically detect mentioned areas of interest, a prerequisite for automated processing. In Multimedia Appendix 1, we present a video sequence showing an anesthesia team managing a critical anesthesia event, providing a good overview of the simulation environment, the patient monitor used, and the eye-tracking footage.

### Data Collection, Video Analysis, and Data Exclusion

Before starting each scenario, we calibrated the eye-tracking device to the participant. We recorded the subject's field of view as a video feed with Pupil Invisible, a mobile eye-tracking device similar in shape and size to regular glasses. The device was connected to a mobile phone that participants carried in their pockets, which served as a power source and storage unit. After each recording, we uploaded the eye-tracking data to a research server and made backup copies on physical hard drives.

For the analysis, we first manually checked the eye-tracking data's quality. Then, for the post hoc semi-automated video analysis, we used Pupil Labs proprietary software Pupil Player on an Acer Aspire V15 Nitro laptop (Acer Inc). Within Pupil Player, we delimited the patient monitor as our area of interest, using the surface tracker plugin. Using the fixations detector plugin and cropping the videos to the start and end of the 10-minute simulation (designated by a bell signal), we were then able to automatically export all fixations and their durations as Microsoft Excel spreadsheets (version 16.58; Microsoft Corporation). During post hoc editing, all recordings were manually stopped five times per recording to ensure the accuracy of the boundaries of the areas of interest. Multimedia Appendix 2 shows an example sequence of the analyzed eye-tracking data in Pupil Player.

### Outcome Measures

As our primary outcome, we defined visual attention as fixation count and dwell time. As a fixation, we counted every instance where the subject’s gaze rested in one single location within the area of interest for more than 100ms. The dwell-time corresponds to the cumulative time in seconds that the participant’s gaze was focused on the predefined area of interest. In addition to visual attention, we collected potentially influencing variables such as screen type (only conventional, only avatar or split-screen monitoring), scenario (bronchospasm, myocardial infarction, or malignant hyperthermia), sequence of the scenarios, profession (nurse anesthetist or anesthesiologist), and work experience (in years). Furthermore, we looked at the relation of our primary outcome and the participant’s task performance. The task performance was based on the time required for participants to perform critical diagnostic and therapeutic tasks during the scenarios [20]. An example of such a therapeutic task in the malignant hyperthermia scenario is stopping the trigger or administrating dantrolene.

### Statistical Analysis

In this exploratory study design, a power calculation was not performed. For descriptive statistics, we report means with standard deviation and medians with IQRs for continuous data and numbers and percentages for categorical data. We used mixed linear models to analyze fixations count and dwell time. In the models, we included the potentially influential variables task performance, screen type, scenario, sequence of the scenarios, profession, and work experience as covariates. We used R version 4.0.5 (R Foundation for Statistical Computing,) to analyze all data and used Prism 9 (GraphPad Software Inc) to create all figures. We considered a *P*-value of less than .05 to determine statistical significance.

## Results

**Figure 2:**
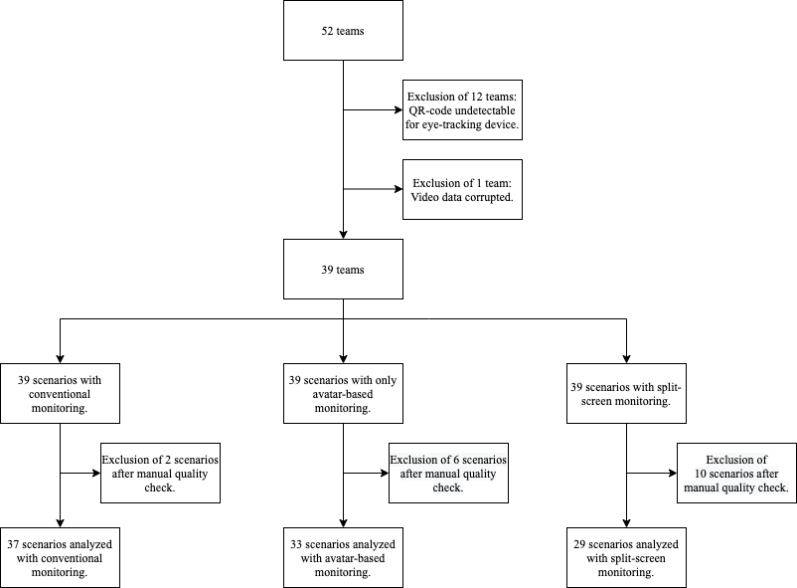
Study setup and exclusion criteria. We analyzed 99 ten-minute scenarios performed by 39 anesthesia teams. Exclusion of 12 teams because the laminated QR codes used reflected and were not detected by the eye-tracking software; exclusion of 1 team because the video footage was incomplete; exclusion after the manual quality check of 18 scenarios due to inaccuracies in eye-tracking calibration (e.g., alternate blinking or wearing of prescription glasses).

**Figure 3:**
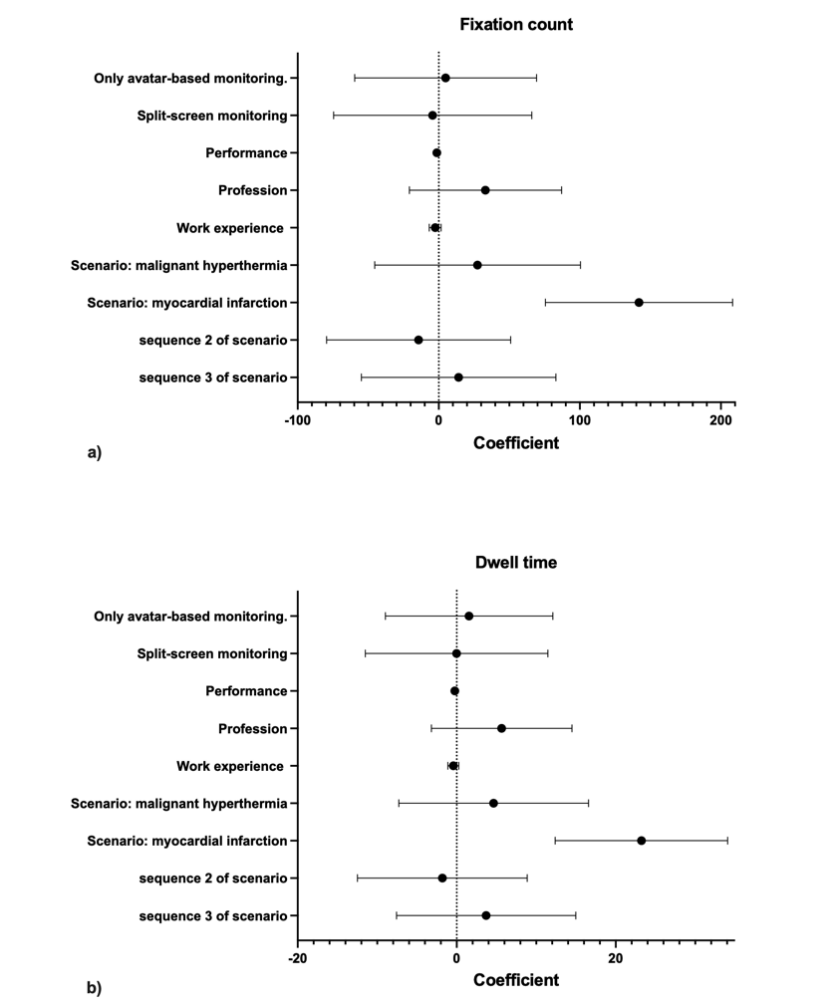
Results for adjusted, mixed linear models a) for fixation counts and b) for dwell time. Both models take the potential influential variables screen type (conventional, only avatar or split-screen monitoring), performance in managing the simulated critical anesthesia events (task performance, in percent), profession (nurse anesthetist or anesthesiologist), work experience (in years), the simulated scenario (bronchospasm, myocardial infarction, or malignant hyperthermia) and the sequence of simulation into account.

In May 2020, we recruited 52 teams performing 156 simulations. We excluded 12 teams as the eye-tracking setup was revised because the laminated QR codes used reflected and were not detected by the eye-tracking software. In addition, we had to exclude another team because the video footage was incompletely recorded. Finally, after a manual quality check of the data, we excluded 18 scenarios due to inaccuracies in eye-tracking calibration (eg, alternate blinking or wearing of prescription glasses). This left us with 99 ten-minute video sequences for the eye-tracking video analysis. Table 1 provides additional study and participant characteristics. Figure 2 shows the study setup and the exclusion criteria of the video footage in more detail, and Figure 3 summarizes our results.

**Table 1:** Study and participant characteristics.

Study characteristics				Values
	Number of simulations conducted, N			156
	**Number of eye tracking analyzed, N**			99
		Screen modalities, n (%)		
		Only conventional monitoring	37 (37%)	
		Only visual-patient-avatar	33 (33%)	
		Split-screen monitoring	29 (30%)	
	**Scenarios, n (%)**			
		Severe Bronchospasm	30 (30%)	
		Myocardial infarction	36 (36%)	
		Malignant hyperthermia	33 (33%)	
**Participant characteristics, N**				39
	**Team leader**			
		Nurse anesthetist	16 (41%)	
		Anesthesiologist	23 (59%)	
	Experience team leader (years), median (IQR)			4 (1.5-8)

### Visual Attention on the Patient Monitor

Overall, participants had a median (IQR) of 198 (93-318) fixations on the monitor with a median (IQR) dwell time of 30.2 (14.9-51.3) seconds. This means that participants spent around 5% of their visual attention on the patient monitor screen during the 10-minute simulation.

### Visual Attention and Screen Modality

Comparing the fixations on the two avatar-based screen modalities with the fixations to conventional monitoring, the mixed linear model yielded a coefficient of 4.89 fixations (95% CI –59.57 to 69.35; *P*=.89) for only avatar-based monitoring and a coefficient of -4.33 fixations (95%CI: –74.55 to 65.90; *P*=.91) for the split-screen modality. This means that participants using only avatar-based monitoring in the simulated scenarios had about five fixations more, and participants using the split-screen modality had about four fixations less than when they used conventional monitoring. However, those results were not statistically significant.

Comparing the dwell time on the avatar-based screen modalities with the dwell time on the conventional monitor using an adjusted, mixed linear model, we found a coefficient of 1.56 seconds (95% CI –8.98 to 12.09; *P*=.78) for only avatar-based monitoring and a coefficient of –0.02 seconds (95% CI –11.50 to 11.46; *P*=1.00) for the split-screen modality. This means that participants using only avatar-based monitoring in the simulated scenarios looked around 1.6 seconds longer on the monitor screen. Participants using the split-screen spent as much time on the patient monitor as those using conventional monitoring. However, those results were also not statistically significant.

### Visual Attention and Task Performance

Regarding task performance, the mixed linear models showed that the fixation count and the dwell time decreased with better task performance regardless of the screen modality used (fixation: coefficient –1.39; 95% CI –2.44 to –0.34; *P*=.02 and dwell time: coefficient –0.23; 95%CI –0.4 to –0.06; *P*=.01). Thus, with each percentage point of better task performance, the number of fixations decreased by about 1.39, and the dwell time diminished by 0.23 seconds.

### Visual Attention and Other Potentially Influencing Factors

Regarding the potentially influential variables, profession, work experience, and sequence of the scenarios, the mixed linear models yielded no evidence for differences in fixations and dwell time (Figure 3). However, the scenarios themselves differed with respect to fixation and dwell time (Figure 3). Participants had significantly more fixations (coefficient 141.97; 95% CI 75.62 to 208.32; *P*<.001) and a higher dwell time (coefficient 23.23; 95%CI 12.48 to 34.08; *P*<.001) on the patient monitor in the myocardial infarction scenario.

### Per-Screen Analysis For Split-Screen Modality

Further, we found that for the conventional half of the split-screen modality, participants had 158 (IQR 63-226) fixations and a dwell time of 24.3 (IQR 10.0-36.8) seconds. For the avatar-based half of the split-screen modality, participants had 44 (IQR 28-84) fixations and a dwell time of 6.8 (IQR 4.3-13.3) seconds. Using a Mann-Whitney test to compare both halves of the split-screen, we found that subjects had significantly fewer fixations (*P*<.001) and significantly less dwell time (*P*=.001) on the avatar part of the patient monitor.

## Discussion

### Overview

This study investigated whether avatar-based patient monitoring influences the visual attention of anesthesia providers on the patient monitor. We assessed 99 eye-tracking videos of anesthesia personnel managing simulated critical anesthesia events. We found no significant difference in visual attention when anesthesia providers used avatar-based or conventional patient monitoring in simulated critical anesthesia events using adjusted, mixed linear models.

### Visual Attention on the Patient Monitor

Anesthesia personnel devoted about 5% of their time to the patient monitor. These results are consistent with findings under real-life conditions [29]. However, other simulation studies reported higher percentages of dwell time on the patient monitor [29,30]. The high fidelity of our simulations may explain these differences. We used an in-situ simulation design and enhanced the simulation’s fidelity further by using real medications and airway management tools in addition to a state-of-the-art, full human patient simulator. Thus, it is conceivable that a very realistic simulation is more likely to align participant behavior with outcomes under real-life conditions than a simulation with lower fidelity.

### Visual Attention and Screen Modality

We hypothesized that the anesthesia provider's visual attention on the patient monitor would decrease with avatar-based patient monitoring due to accelerated and simplified information transfer found in the previous studies [17-19,26,27]. The basic idea behind this hypothesis is that the qualitative visualization of the patient avatar may lead to a quicker overview of the patient's situation [27,31]. In addition, avatar-based patient monitoring highlights pathophysiological changes, eliminating the time-consuming task of creating a mental model from the various numerical values of the conventional patient monitor [5,6,32]. This may speed up the perception of the situation, lead to fewer fixations and less dwell time on irrelevant vital signs and consequently lead to less visual attention on the patient monitor. In other words, the anesthesia provider knows where to look and can therefore perceive the necessary information with less visual attention. However, this eye-tracking study did not confirm our hypothesis. We found no significant difference in participants' visual attention when using avatar-based monitoring compared to conventional patient monitoring. Unfamiliarity with the novel, avatar-based technology may have masked its potential benefits and may serve as a possible explanation for the finding as all participants used the visual-patient-avatar for the first time. Benefits and acceptance of a new technology depend heavily on users' exposure [33].

However, we found significant differences between the two halves of the split-screen modality. Participants had significantly more visual attention on the conventional part than on the avatar part of the split-screen monitor. This may indicate an interaction effect after all. Perhaps the avatar drew their attention to something (eg, a vital sign outside the norm) that they checked on the conventional screen. Because the qualitative visualization provided by the avatar is intuitive and quickly understood [27,31], participants paid relatively little visual attention to the avatar. To verify the qualitative input, participants had to extract information from the various numbers and waveforms on the conventional screen, a time-consuming task [5,6,32].

Qualitative data collection on how participants used the patient monitors might have helped clarify the result concerning the split-screen modality. Mixed methods are often more powerful than purely quantitative data analysis for such complex human factors work [34].

### Visual Attention, Task Performance, and Situation Awareness

**Figure 4:**
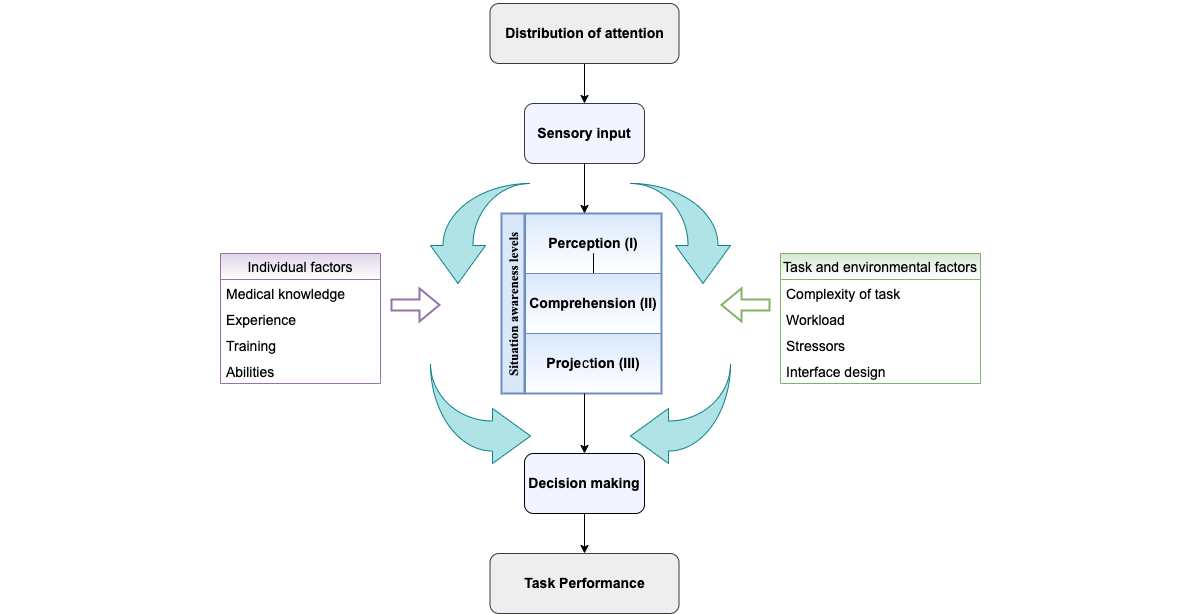
Illustration of situation awareness in the context of health care. (Adapted from Schulz, C.M. et al., Situation Awareness in Anesthesia: Concept and Research. Anesthesiology 2013; 118:729–742 and Endsley, M.R., Towards a theory of situation awareness in dynamic systems. Hum Factors 1995; 37:32–64) The framework illustrates that adequate situation awareness is a prerequisite for informed decision-making. The acquisition of situational awareness starts with the perception of sensory inputs (mainly visual and auditive). The inputs must be understood, and based on that understanding, a projection must be made on the present and future of the situation. Now good decision-making can occur, leading to good task performance in the clinical context. Individual, task, and environmental factors may influence all levels of situation awareness. As a situation changes over time, a continuous reevaluation is obligatory to maintain adequate situation awareness.

We found that an increase in the anesthesia team’s task performance was associated with decreased visual attention. The correlation between the two parameters supports the notion that visual attention and task performance act as indirect indicators of situation awareness [4,30,35-38]. The distribution of visual attention determines what is in the perceptual field and, therefore, contributes to the sensory input, an essential aspect of perception (ie, situation awareness level I) [32]. Good clinical performance in managing the simulated critical anesthesia events comes at the end of good decision-making [38,39], which requires a sufficient understanding (ie, situation awareness level II) and a projection of the situation's near future (ie, situation awareness level III) [32]. A combination of eye tracking and performance measures simultaneously determined what information had been seen and to what degree this information had been perceived and comprehended by the anesthesia provider, giving us a good idea about all three levels of situation awareness achieved (Figure 4).

This study may contribute exciting aspects to the current debate on how to best measure situation awareness [4,30,40-42]. Questionnaires to be used during simulations were proposed and validated as direct measurement tools for situation awareness [4,40]. These direct measurement methods require pausing the simulation to answer the questionnaire before resuming the task again [40]. Evidently, this instrument for assessing situation awareness has limited application in clinical reality, as there is no time to stop the treatment of a critical patient to interview the treating physician. For this reason, we propose the combination of the indirect measurement parameters visual attention and task performance as a surrogate when measurements of situation awareness in clinical praxis are wanted.

### Visual Attention and Other Potentially Influencing Factors

The three simulated emergencies received different amounts of visual attention. In the myocardial infarction scenario, participants looked at the patient monitor more frequently and had a higher dwell time (Figure 3). In this simulation, there was the additional option of displaying a 12-lead electrocardiogram on the patient monitor, necessary to diagnose myocardial infarction. Although we manually cropped the 12-lead electrocardiogram sequence because we did not analyze it in the context of conventional patient monitoring or avatar-based patient monitoring, participants may have searched for a relatively long time on the monitor to activate the 12-lead electrocardiogram function. This circumstance may explain the significantly increased visual attention on the patient monitor in this scenario.

Furthermore, we found a tendency (not significant) that anesthesia nurses had more fixations and a higher dwell time on the patient monitor (Figure 3). Anesthesia nurses perform important preparatory tasks but generally spend less time with direct anesthesia management. This circumstance may result in anesthesia nurses paying slightly more visual attention to the patient monitor to gain the same level of situational awareness as an anesthesiologist whose main task is anesthesia management.

### Strengths and Limitations

Our study had several limitations. First, we had to exclude more than one-third of all simulations from our analysis due to technical issues or poor data quality of the eye-tracking footage. Although we used one of the latest mobile eye-tracking devices on the market, we faced several challenges while recording the data: calibrating the glasses for ocular pathologies (eg, alternating squinting or wearing prescription glasses), battery-life issues, or the device slipping of the participants face during physical tasks (eg, manual resuscitation). This shows that despite the massive development of eye-tracking hardware and software in recent years, the technology is still error-prone. Second, all study participants were unfamiliar with avatar-based patient monitoring. Therefore, the results may vary as anesthesia personnel becomes accustomed to this new technology. Third, the median work experience of our participants is relatively low at four years. Finally, we conducted this study in a university hospital in Europe. Therefore, results may differ under other conditions and in other parts of the world.

The study had several strengths. First, we reduced selection bias by balanced participant selection and consistent randomization of the scenario sequence, team leader, and screen-modality. Second, a rigorous manual quality check of the eye-tracking recordings ensured excellent data quality, allowing us to replace error-prone manual eye-tracking analysis with automated analysis. Third, we attempted to circumvent the bias of authenticity inherent in all simulation-based studies [43] through our in-situ study design and our efforts to represent clinical reality as accurately as possible in this high-fidelity simulation study. Finally, we used eye-tracking hardware that was no more distracting than wearing regular glasses to produce high-quality video footage.

### Conclusions

We found no significant difference in visual attention when anesthesiologists used the novel avatar-based or the conventional patient monitoring in simulated critical anesthesia events. However, when using the split-screen displaying the conventional monitoring alongside the avatar-based monitoring, significantly less visual attention was paid to the avatar side of the screen. This may indicate an interaction effect. Perhaps the avatar drew the participant's attention to a vital sign outside the norm that they checked on the conventional screen. Because the qualitative visualization provided by the avatar is intuitive and quickly understood, participants paid relatively little visual attention to the avatar. To verify the qualitative input on the conventional monitor screen seems to have taken more time. In addition, we identified visual attention in conjunction with task performance as a valuable surrogate for situational awareness as it covers all three levels of situational awareness.

## Appendix

Multimedia Appendix 1 Video sequence of an anesthesia team solving a simulated critical anesthesia event, providing a good overview of the simulation environment, the patient monitor used, and the eye-tracking footage.

Multimedia Appendix 2 Example sequence of the analyzed eye-tracking data.

## References

[1] (2009). WHO guidelines for safe surgery 2009: safe surgery saves lives. https://apps.who.int/iris/handle/10665/44185.

[2] Fletcher G, McGeorge P, Flin R, Glavin R, Maran N (2002). The role of non-technical skills in anaesthesia: a review of current literature. Br J Anaesth.

[3] Fioratou E, Flin R, Glavin R, Patey R (2010). Beyond monitoring: distributed situation awareness in anaesthesia. Br J Anaesth.

[4] Schulz CM, Endsley MR, Kochs EF, Gelb AW, Wagner KJ (2013). Situation awareness in anesthesia: concept and research. Anesthesiology.

[5] Miller GA (1956). The magical number seven, plus or minus two: Some limits on our capacity for processing information. Psychological Review.

[6] Drews FA, Westenskow DR (2006). The right picture is worth a thousand numbers: data displays in anesthesia. Hum Factors.

[7] Ruskin K, Hueske-Kraus Dirk (2015). Alarm fatigue: impacts on patient safety. Curr Opin Anaesthesiol.

[8] Waller R, Wright MC, Segall N, Nesbitt P, Reese T, Borbolla D, Del Fiol G (2019). Novel displays of patient information in critical care settings: a systematic review. J Am Med Inform Assoc.

[9] McFarlane DC, Doig AK, Agutter JA, Mercurio JL, Mittu R, Brewer LM, Syroid ND (2016). Defeating information overload in health surveillance using a metacognitive aid innovation from military combat systems. Journal of Defense Modeling & Simulation.

[10] Schulz CM, Burden A, Posner KL, Mincer SL, Steadman R, Wagner KJ, Domino KB (2017). Frequency and Type of Situational Awareness Errors Contributing to Death and Brain Damage: A Closed Claims Analysis. Anesthesiology.

[11] Long J, Jowsey T, Garden A, Henderson K, Weller J (2020). The flip side of speaking up: a new model to facilitate positive responses to speaking up in the operating theatre. Br J Anaesth.

[12] Arriaga Alexander F, Bader Angela M, Wong Judith M, Lipsitz Stuart R, Berry William R, Ziewacz John E, Hepner David L, Boorman Daniel J, Pozner Charles N, Smink Douglas S, Gawande Atul A (2013). Simulation-based trial of surgical-crisis checklists. N Engl J Med.

[13] Wright M, Dunbar S, Macpherson B, Moretti E, Del Fiol G, Bolte J, Taekman J, Segall N (2017). Toward Designing Information Display to Support Critical Care. Appl Clin Inform.

[14] McNeer RR, Horn DB, Bennett CL, Edworthy JR, Dudaryk R (2018). Auditory Icon Alarms Are More Accurately and Quickly Identified than Current Standard Melodic Alarms in a Simulated Clinical Setting. Anesthesiology.

[15] Roche TR, Braun J, Ganter MT, Meybohm P, Herrmann J, Zacharowski K, Raimann FJ, Piekarski F, Spahn DR, Nöthiger CB, Tscholl DW, Said S (2021). Voice alerting as a medical alarm modality for next-generation patient monitoring: a randomised international multicentre trial. Br J Anaesth.

[16] Tscholl DW, Rössler Julian, Said S, Kaserer A, Spahn DR, Nöthiger Christoph Beat (2020). Situation Awareness-Oriented Patient Monitoring with Visual Patient Technology: A Qualitative Review of the Primary Research. Sensors (Basel).

[17] Tscholl D, Handschin L, Neubauer P, Weiss M, Seifert B, Spahn D, Noethiger C (2018). Using an animated patient avatar to improve perception of vital sign information by anaesthesia professionals. Br J Anaesth.

[18] Garot O, Rössler J, Pfarr J, Ganter MT, Spahn DR, Nöthiger CB, Tscholl DW (2020). Avatar-based versus conventional vital sign display in a central monitor for monitoring multiple patients: a multicenter computer-based laboratory study. BMC Med Inform Decis Mak.

[19] Pfarr J, Ganter MT, Spahn DR, Noethiger CB, Tscholl DW (2019). Avatar-Based Patient Monitoring With Peripheral Vision: A Multicenter Comparative Eye-Tracking Study. J Med Internet Res.

[20] Roche TR, Said S, Braun J, Maas EJ, Machado C, Grande B, Kolbe M, Spahn DR, Nöthiger CB, Tscholl DW (2021). Avatar-based patient monitoring in critical anaesthesia events: a randomised high-fidelity simulation study. Br J Anaesth.

[21] Tscholl DW, Weiss M, Handschin L, Spahn DR, Nöthiger Christoph B (2018). User perceptions of avatar-based patient monitoring: a mixed qualitative and quantitative study. BMC Anesthesiol.

[22] Rössler J, Kaserer A, Albiez B, Braun J, Breckwoldt J, Spahn DR, Nöthiger CB, Tscholl DW (2020). Comparing Classroom Instruction to Individual Instruction as an Approach to Teach Avatar-Based Patient Monitoring With Visual Patient: Simulation Study. JMIR Med Educ.

[23] Endsley MR (2016). Measurement of Situation Awareness in Dynamic Systems. Hum Factors.

[24] de Winter JCF, Eisma YB, Cabrall CDD, Hancock PA, Stanton NA (2018). Situation awareness based on eye movements in relation to the task environment. Cogn Tech Work.

[25] Harezlak K, Kasprowski P (2018). Application of eye tracking in medicine: A survey, research issues and challenges. Comput Med Imaging Graph.

[26] Pfarr J, Ganter MT, Spahn DR, Noethiger CB, Tscholl DW (2020). Effects of a standardized distraction on caregivers' perceptive performance with avatar-based and conventional patient monitoring: a multicenter comparative study. J Clin Monit Comput.

[27] Tscholl DW, Rössler J, Handschin L, Seifert B, Spahn DR, Nöthiger CB (2020). The Mechanisms Responsible for Improved Information Transfer in Avatar-Based Patient Monitoring: Multicenter Comparative Eye-Tracking Study. J Med Internet Res.

[28] Urbaniak GC, Plous S Research Randomizer (Version 4.0).

[29] Grundgeiger T, Klöffel C, Mohme S, Wurmb T, Happel O (2017). An investigation into the effects of real vs. stimulated cases and level of experience on the distribution of visual attention during induction of general anaesthesia. Anaesthesia.

[30] Schulz C, Schneider E, Fritz L, Vockeroth J, Hapfelmeier A, Brandt T, Kochs E, Schneider G (2011). Visual attention of anaesthetists during simulated critical incidents. Br J Anaesth.

[31] Lin YL, Trbovich P, Kolodzey L, Nickel C, Guerguerian A (2019). Association of Data Integration Technologies With Intensive Care Clinician Performance: A Systematic Review and Meta-analysis. JAMA Netw Open.

[32] Endsley MR, Bolte B, Jones DG (2003). Designing for Situation Awareness: An Approach to User-Centered Design. https://www.taylorfrancis.com/books/mono/10.1201/9780203485088/designing-situation-awareness-mica-endsley-betty-bolte-debra-jones.

[33] Dziadzko MA, Herasevich V, Sen A, Pickering BW, Knight AA, Moreno Franco P (2016). User perception and experience of the introduction of a novel critical care patient viewer in the ICU setting. Int J Med Inform.

[34] Webster CS (2019). Evidence and efficacy: time to think beyond the traditional randomised controlled trial in patient safety studies. Br J Anaesth.

[35] Schulz CM, Schneider E, Fritz L, Vockeroth J, Hapfelmeier A, Wasmaier M, Kochs E, Schneider G (2011). Eye tracking for assessment of workload: a pilot study in an anaesthesia simulator environment. Br J Anaesth.

[36] Liang N, Yang J, Yu D, Prakah-Asante KO, Curry R, Blommer M, Swaminathan R, Pitts BJ (2021). Using eye-tracking to investigate the effects of pre-takeover visual engagement on situation awareness during automated driving. Accid Anal Prev.

[37] Durso FT, Hackworth CA, Truitt TR, Crutchfield J, Nikolic D, Manning CA (1998). Situation Awareness as a Predictor of Performance for En Route Air Traffic Controllers. Air Traffic Control Quarterly.

[38] Hogan MP, Pace DE, Hapgood J, Boone DC (2006). Use of human patient simulation and the situation awareness global assessment technique in practical trauma skills assessment. J Trauma.

[39] Endsley MR (1988). Design and Evaluation for Situation Awareness Enhancement. Proceedings of the Human Factors Society Annual Meeting.

[40] Endsley MR (2021). A Systematic Review and Meta-Analysis of Direct Objective Measures of Situation Awareness: A Comparison of SAGAT and SPAM. Hum Factors.

[41] Salmon PM, Stanton NA, Young KL (2012). Situation awareness on the road: review, theoretical and methodological issues, and future directions. Theoretical Issues in Ergonomics Science.

[42] Chiappe D, Rorie RC, Morgan CA, Vu KL (2012). A situated approach to the acquisition of shared SA in team contexts. Theoretical Issues in Ergonomics Science.

[43] Kurup V, Matei V, Ray J (2017). Role of in-situ simulation for training in healthcare: opportunities and challenges. Curr Opin Anaesthesiol.

